# Role of Neuroendocrine, Immune, and Autonomic Nervous System in Anorexia Nervosa-Linked Cardiovascular Diseases

**DOI:** 10.3390/ijms21197302

**Published:** 2020-10-02

**Authors:** Nikola Sekaninova, Lucia Bona Olexova, Zuzana Visnovcova, Igor Ondrejka, Ingrid Tonhajzerova

**Affiliations:** 1 Department of Physiology, Jessenius Faculty of Medicine in Martin, Comenius University in Bratislava, Mala Hora 4C, 03601 Martin, Slovakia; sekaninova1@uniba.sk (N.S.); olexova10@uniba.sk (L.B.O.); zuzana.visnovcova@uniba.sk (Z.V.); 2 Biomedical Center Martin, Jessenius Faculty of Medicine in Martin, Comenius University in Bratislava, Mala Hora 4C, 03601 Martin, Slovakia; 3 Psychiatric Clinic, Jessenius Faculty of Medicine in Martin, Comenius University in Bratislava, University Hospital Martin, Kollarova 2, 03659 Martin, Slovakia; igor.ondrejka@uniba.sk

**Keywords:** anorexia nervosa, neuroendocrine dysregulation, cytokines, heart rate and blood pressure variability, cardiovascular diseases

## Abstract

Anorexia nervosa represents a severe mental disorder associated with food avoidance and malnutrition. In patients suffering from anorexia nervosa, cardiovascular complications are the main reason leading to morbidity and mortality. However, the origin and pathological mechanisms leading to higher cardiovascular risk in anorexia nervosa are still unclear. In this aspect, the issue of exact pathological mechanisms as well as sensitive biomarkers for detection of anorexia nervosa-linked cardiovascular risk are discussed. Therefore, this review synthesised recent evidence of dysfunction in multiple neuroendocrine axes and alterations in the immune system that may represent anorexia nervosa-linked pathological mechanisms contributing to complex cardiovascular dysregulation. Further, this review is focused on identification of non-invasive biomarkers for the assessment of increased cardiovascular risk in anorexia nervosa that can be linked to a clinical application. Complex non-invasive assessment of cardiovascular autonomic regulation—cardiac vagal control (heart rate variability), sympathetic vascular activity (blood pressure variability), and cardiovascular reflex control (baroreflex sensitivity)—could represent a promising tool for early diagnosis, personalized therapy, and monitoring of therapeutic interventions in anorexia nervosa particularly at a vulnerable adolescent age.

## 1. Introduction

Anorexia nervosa (AN) is an eating disorder defined as abnormally low body weight associated with intense fear of gaining weight and distorted cognition regarding weight, shape, and drive for thinness [1]. The highest incidence rate subgroup for AN includes adolescent girls aged 15–19 years, which accounts for about 40% of all identified cases [2]. Moreover, patients suffering from AN are characterised by neuroendocrine, immune, and autonomic nervous system (ANS) dysregulation resulting in cardiovascular complications and potentially leading to increased morbidity and mortality [3,4,5]. Cardiovascular complications occur in up to 80% of patients with AN, and account for up to 30% of mortality [6]. For example, sinus bradycardia (i.e., resting heart rate less than 50 beats per minute), relatively low arterial blood pressure (hypotension usually lower than 100/50 mmHg), prolonged QT interval, atherosclerosis, etc. are associated with AN even at adolescent age [5]. Despite the fact that some of these changes could represent adaptation to poor nutrition, the origin and pathogenesis are not absolutely clear (according to reviews by Mazurak et al. [7] and Giovinazzo et al. [5]). Thus, we suggest that the issue of precise and sensitive cardiovascular risk assessment is crucial, especially in adolescence.

The aims of this review were (1) to summarize neuroendocrine, immune, and autonomic nervous system pathological mechanisms that contribute to higher cardiovascular risk in AN and (2) to identify potential non-invasive biomarkers for assessment of increased AN-linked cardiovascular risk, representing a promising tool for personalized therapy.

## 2. Neuroendocrine System—The Role in AN-Linked Cardiovascular Diseases

Anorexia nervosa, a condition of profound undernutrition, is associated with global dysregulation in multiple neuroendocrine axes (hypothalamic–pituitary–adrenal/–gonadal/–thyroid axis, growth hormone/insulin-like growth factor-1 axis), and disturbances in appetite-regulating hormones and adipokines. Although most of these disturbances are an adaption to the low energy state as a consequence of chronic starvation and are reversible with an appropriate treatment, neuroendocrine alterations can exert deleterious effects on cardiovascular functioning (e.g., [3,8]).

### 2.1. Anorexia Nervosa-Linked Hypothalamic–Pituitary–Adrenal (HPA) Axis Dysfunction

The hypothalamic–pituitary–adrenal (HPA) axis describes complex mechanisms modulating various physiological processes such as the body’s response to stress, glucose metabolism, and immune functioning mediated by release of key regulatory molecules, corticotropin-releasing hormone (CRH), adrenocorticotropic hormone (ACTH), and cortisol, to maintain homeostasis [9]. Stress has been shown as a potential factor contributing to the development and progression of eating disorders, including anorexia nervosa [10]. Continuous stress of nutritional deprivation in AN leads to chronic HPA axis stimulation, resulting in hypercortisolaemia [11]. Additionally, patients suffering from eating disorders show a blunted HPA axis reactivity to stress exposure resulting in AN-linked health complications [10]. Prolonged dysregulation of the HPA axis associated with enhanced glucocorticoid production is also linked to adverse cardiovascular functioning. However, whether cardiovascular health complications arise from the direct deleterious effects of stress-related HPA axis activation or develop secondarily from the accompanying metabolic changes leading to glucocorticoids excess is still under intensive debate [12].

### 2.2. Abnormalities in the Hypothalamic–Pituitary–Gonadal (HPG) Axis Related to Anorexia Nervosa 

AN-linked dysregulation of the hypothalamic–pituitary–gonadal (HPG) axis is characterised by functional hypothalamic amenorrhea (FHA) associated with relative oestrogen and androgen deficiency leading to anovulation and infertility. FHA results from suppression of gonadotropin releasing hormone (GnRH) in the hypothalamic–pituitary–ovarian axis leading to decreased release of the follicle stimulating hormone (FSH) and luteinizing hormone (LH) from the anterior pituitary, and subsequently reduced oestradiol production. Therefore, endometrial thickening does not occur during the follicular phase, resulting in amenorrhea [13].

Hypoestrogenemia associated with menstrual cycle irregularities in young women leads to increased risk of cardiovascular diseases (CVD). The Nurses’ Health Study of over 82,000 women demonstrated that the more irregular the menstrual cycle is, the greater the risk for future CVD [14]. Moreover, menstrual cycle irregularities have been linked to accelerated uterine atherosclerosis leading to early menopause (defined as menopause ≤45 years old), which is associated with increased cardiovascular morbidity [13].

### 2.3. Hypothalamic–Pituitary–Thyroid (HPT) Axis Dysregulation in Anorexia Nervosa

The hypothalamic–pituitary–thyroid (HPT) axis is responsible for the regulation of metabolism, fluid balance, cardiovascular system functioning, and responding to stress through the production of thyroid hormones [15]. In this aspect, the thyroid hormones play a crucial role in the cardiovascular system homeostasis by stimulating diastolic relaxation and systolic contraction in the myocardium and have a pro-angiogenic effect. However, AN-linked severe weight loss is associated with non-thyroidal illness syndrome characterised by abnormalities in thyroid function tests. Specifically, the level of total triiodothyronine (T3) is low (an adaptive mechanism to lower resting energy expenditure and conserving energy for vital functions), reverse T3 is elevated (increased peripheral deiodination of thyroxine (T4) to reverse T3), and free T4 and thyroid stimulating hormone vary from normal to low-to-normal [16]. These AN-linked abnormalities normalize during weight gain [16], but long-term thyroid axis dysfunction is associated with impaired myocardial bioenergetic status that may contribute to increased CVD incidence [17].

### 2.4. Growth Hormone (GH)/Insulin-Like Growth Factor 1 (Igf-1) Axis

Growth hormone (GH) represents a proteohormone secreted by the pituitary gland that is involved in metabolic functions. Specifically, GH via stimulating insulin-like growth factor 1 (IGF-1) production increases the concentration of glucose and free fatty acids [18]. Moreover, the GH/IGF-1 axis contributes to normal cardiovascular functioning—stimulation cardiac growth, contractility, and regulation of the vascular tone and peripheral resistance. Thus, prolonged GH excess as well as GH deficiency are associated with increased cardiovascular morbidity [19]. From this aspect, AN is associated with acquired GH resistance: increased GH secretion but decreased IGF-1 levels. Several mechanisms of GH resistance in AN have been proposed: increased fibroblast growth factor 21 inhibiting STAT-5, a mediator of intracellular GH effects; low levels of insulin due to downregulation of the expression of hepatic GH receptors; or increased levels of ghrelin leading to stimulation of GH secretion [3].

### 2.5. Adipokines And Appetite-Regulating Hormones—Leptin, Adiponectin, Ghrelin, Peptide YY (PYY)

Leptin, an anorexigenic adipokine secreted by adipose tissue, play an important role in body weight homeostasis and psychophysiological processes that are associated with AN [20]. Leptin has been shown to exert pleiotropic effects by influencing haematopoiesis, thermogenesis, reproduction, angiogenesis, and most importantly neuroendocrine and immune homeostasis. In this context, leptin can influence HPA axis by regulating the secretion of HPA hormones in the hypothalamus. Leptin regulates the minute-to-minute oscillations in the luteinizing hormone and oestradiol levels. Nocturnal leptin increase determines the change in the nocturnal luteinizing hormone profile in the mid-to-late follicular phase that precedes ovulation. In this context, decrease in circulating levels of leptin is probably responsible for the disruption of HPG function (reduction in LH oscillations and hence oestrogen deficiency) in AN patients [21]. Moreover, leptin can affect innate and adaptive immunity by inducing a proinflammatory response [22]. Serum leptin levels have been repeatedly found to be decreased in AN patients compared to that of controls (e.g., [23,24]), however, it seems to be reversible, and leptin levels increase during weight recovery [25].

Adiponectin represents the most abundant peptide secreted by adipocytes, playing an important role in the metabolism, regulation of anabolic pathways, reduction of oxidative stress, prevention from inflammatory processes, and vascular function improvement [26]. Misra et al. [27] reported unchanged levels of adiponectin in AN women compared to that of normal-weight controls, however, other studies revealed elevated adiponectin levels [28] or decreased adiponectin levels (e.g., [29]). Thus, further research on this issue is needed. 

Ghrelin is a centrally acting appetite-stimulating peptide produced by multiple organs (e.g., endocrine cells in the stomach, pancreas, intestine, etc.) implicated in various functions. Specifically, ghrelin has stimulatory effects on food intake, gastrointestinal motility, lipogenesis, and blood glucose levels and inhibitory effects on blood pressure and LH and FSH release [30]. Impairments in ghrelin secretion may play an important role in the development of anorexia nervosa [31]. Several studies reported increased plasma levels of ghrelin in AN patients [32]. Moreover, elevated ghrelin levels, which usually decrease with weight recovery, are inversely correlated with body mass index (BMI) [33]. Notably, impaired ghrelin signalling and modulation indexed by a delayed or absent postprandial ghrelin decrease [34], the inability to adequately suppress the secretion of growth hormone after glucose digestion [35], or the insufficiency of glucose elevation after exogenous ghrelin application [36] suggest AN-linked ghrelin resistance [30].

Peptide YY (PYY) as an anorexigenic hormone that supresses appetite and is secreted by intestinal L cells. Serum levels of PYY are shown to be increased in AN patients compared to that of controls, contributing to decreased nutrient intake and disordered eating psychopathology [37].

Dysregulation in appetite-regulating hormones and adipokines may contribute to increased risk of CVD. Specifically, experimental studies suggested that leptin deficiency contributes to cardiac contractile dysfunction, impaired intracellular Ca^2+^ homeostasis, and ultrastructural derangement in ventricular myocytes [38]; decreased adiponectin levels are associated with the development of obesity and subsequent adverse cardiovascular functioning [39]; pathophysiological concentration of ghrelin was shown to increase the expression of endothelial adhesion molecules involved in vascular inflammation [40]; and PYY could adversely affect cardiac structure/function by activating cardiac fibroblasts [41].

## 3. Immune System Abnormalities Related to Cardiovascular Risk in Anorexia Nervosa

The important role of the immune system in the pathogenesis of various diseases, including mental diseases, is being increasingly accepted. However, the influence of inflammation in the development and maintenance of anorexia nervosa is still under intensive debate. Importantly, functioning of the immune system is controlled by the neuroendocrine system. In this context, the above mentioned AN-linked hormonal imbalances may result in uncontrolled immune system changes (e.g., altered cytokines production) and, vice versa, released cytokines can influence neuroendocrine functioning via their direct action on the brain [42,43]. Potential contributors to the dysregulated immune system (proinflammatory state) in AN include increased oxidative stress, a stress-related chronically activated HPA axis and sympathetic nervous system, and changes in the intestinal microbiota, all of which are present in anorexia nervosa [44].

### 3.1. Cytokines—Their Role in AN Psychopathology

Cytokines represent a broad group of secreted proteins that are important in cell signalling. These messenger molecules include chemokines, interferons (IFN), interleukins (IL), lymphokines, and tumour necrosis factors (TNF). Cytokines as intercellular signalling molecules with particular importance in the immune system are supposed to play a mediatory role in the complex neuroendocrine–immune relationship [43]. From a psychoimmunological aspect, the cytokines are divided according to their immunological function into four categories [45]:

(1) T_H_1 cytokines (IL-2, IL-12, IFN-γ) promoting the T_H_1 branch of the immune system and leading to cytotoxic cell contacts,
(2) T_H_2 cytokines (IL-4, IL-5, IL-13) stimulating the T_H_2 branch and induction of antibodies production,
(3) The proinflammatory cytokines (IL-1, IL-6, IL-8, IL-17, IL-21, IL-22, IFN-α, TNF-α) that promote inflammation,
(4) The anti-inflammatory cytokines (IL-10, TGF-ß) that are influenced by regulatory T cells, preventing inflammatory processes.

Recent meta-analysis has shown that AN is associated with elevated levels of certain proinflammatory cytokines including TNF-α and IL-6 [43]. In addition, elevated IL-6 serum concentrations in AN normalize during the twelve weeks of specialised AN treatment [46].

From a psychophysiological perspective, cytokines have been implicated in emotional/cognitive regulation. Specifically, cytokines produced in the body’s periphery can access the brain via humoral, neural and cellular pathways, influencing the mental state through the modulation of neurotransmitters metabolism and signal transduction, modulation of the HPA axis, induction of appetite-regulating hormones release, and impact on neural plasticity and neurogenesis [42,43]. Moreover, a proinflammatory state in the periphery may lead to chronic neuroinflammation. Specifically, peripherally synthesized TNF-α may cause microglial activation and subsequent proinflammatory expression (e.g., TNF-α, IL-1β, etc.) in the brain [47] leading to an amplification of the neuroinflammatory response with detrimental effects on neural, cognitive, and behavioural functions [48]. Thus, altered cytokines (e.g., TNF-α) production associated with chronic neuroinflammation may contribute to mood and cognitive impairments [49,50] that are commonly reported in AN patients.

### 3.2. Cytokines—The Role in AN-Linked Increased Cardiovascular Risk

Generally, primary mediators of inflammation are macrophage-derived cytokines, e.g., IL-1β and TNF-α. These cytokines activate nuclear factor κB, which in turn increases the production of IL-6 and IL-8 and induces T cells to produce IFN-γ [51]. Notably, inflammation is considered to play a key role in increased CVD risk [52]. Specifically, increased production of cytokines, chemokines, and endothelial adhesion molecules were observed in affected cardiac tissues [53], thus, altered cytokines production may contribute to the inflammation-linked increased risk of CVD in AN patients. From this point-of-view, persistent TNF-α-activated signal transduction pathways are associated with vascular dysfunction, atherogenesis, hypertension, and adverse cardiac remodelling [54]; both chronically elevated IL-6 levels and IL-6 receptor protein overexpression may lead to continuous activation of glycoprotein 130, resulting in myocardiac hypertrophy [55].

**Figure 1:**
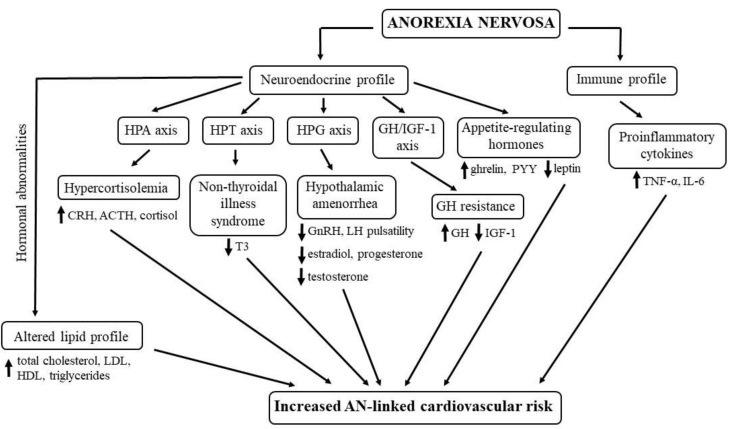
The complex neuroendocrine–immune pathways leading to a higher risk of cardiovascular diseases in anorexia nervosa. HPA, hypothalamic–pituitary–adrenal; HPT, hypothalamic–pituitary–thyroid; HPG, hypothalamic–pituitary–gonadal; GH, growth hormone; IGF-1, insulin-like growth factor 1; CRH, corticotropin-releasing hormone; ACTH, adrenocorticotropic hormone; T3, triiodothyronine; GnRH, gonadotropin-releasing hormone; LH, luteinizing hormone; PYY, peptide YY; TNF-α, tumour necrosis factor α; IL-6, interleukin 6; LDL, low-density lipoprotein; HDL, high-density lipoprotein; AN, anorexia nervosa; up-arrow, increased levels; down-arrow, decreased levels.

The complex neuroendocrine–immune dysregulation associated with AN-linked increased cardiovascular risk is summarized in Figure 1.

In terms of cardiovascular risk, previous studies referred to a connection between the autonomic nervous system (ANS) and the immune system - “the cholinergic anti-inflammatory pathway” - that represents a neural inhibitory mechanism of the peripheral release of TNF, IL-1, and other proinflammatory cytokines through parasympathetic (vagal) outflow [56,57]. Recently, this model has been modified to the sympathetic-cholinergic anti-inflammatory pathway, which emphasizes the role of the sympathetic output in the anti-inflammatory response [58]. It seems that future research would be interesting to detect ANS non-invasive biomarkers with respect to the inflammatory profile for early diagnosis of cardiovascular diseases and personalized therapy in anorexic patients.

## 4. Autonomic Nervous System Dysregulation as A Potential Mechanism Leading to Cardiovascular Diseases in Anorexia Nervosa

The autonomic nervous system plays a crucial role in the maintenance of homeostasis. Both divisions—parasympathetic and sympathetic—are tonically active, and their close cooperation is known as a dynamic sympathovagal balance. Importantly, proper sympathovagal balance functioning at rest and in response to stress is important for organism flexibility, adaptability, and physical/mental health. In contrast, a lack of dynamic adaptability characterized by autonomic imbalance (i.e., the sympathetic or parasympathetic nervous system dominates over the other) is associated with a higher risk of cardiovascular and other health complications [59,60,61]. In addition, inter-individual differences in the stress response system to long-term stress exposition associated with altered autonomic regulation may contribute to the risk for development of mental and other disorders [62].

### 4.1. Heart Rate Variability—An Index of Cardiac Vagal Regulation 

Cardiac function is extremely sensitive to autonomic regulatory inputs. With respect to cardiac vagal control, Mazurak et al. [7] in their review identified three distinct responses of cardiac vagal control in AN: a dominance of parasympathetic activity (e.g., [63,64]), lower cardiovagal control (e.g., [65]), or no differences (e.g., [66]). Although the majority of the studies indicated parasympathetic dominance as an adaptive response to conserve energy and caloric deprivation [67,68,69], other mechanisms should be taken into account. Recent neuroimaging studies revealed anorexia-linked abnormalities in central brain structures (e.g., reductions in subcortical volumes, cortical thickness), or the neural alteration of AN-linked reward processing, suggesting dysfunctions in fronto-striatal and insular brain regions [70,71,72,73,74]. Moreover, Gorwood et al. [75] proposed that AN results from dysregulations of regulatory centres involved in the balance between input (feeding/hunger) and output (excessive exercise), including genetic and epigenetics factors (e.g., dopamine genes involved in the reward circuitry located in the ventral striatum and the food regulatory mechanisms located in the hypothalamus). These brain structures overlap with regions of the central autonomic regulatory network of the heart rate (HR).

Mean HR is determined by dynamic interaction of acceleratory sympathetic nervous activity (especially to stress), and dominant deceleratory parasympathetic nervous system activity results in rhythmical beat-to-beat oscillations—heart rate variability (HRV) [60]. With respect to neurocardiac integrity, Benarroch [76] described the central autonomic network (CAN) as an integrated regulatory mechanism through which the brain controls complex visceromotor, neuroendocrine, and behavioural responses. Structural cortical and subcortical components of CAN (e.g., prefrontal cortex, amygdala) are included in the regulation of sympathetic and parasympathetic outputs to sinoatrial nodes, producing the complex HRV indicative of a healthy and adaptive organism [60,77]. In contrast, rigid system functioning without brisk adaptation to stress results in low HRV that is associated with higher risk of morbidity and mortality [60,78]. Thus, the autonomic imbalance might be the final common pathway linking the disorders and conditions to death and disease [79]. From this perspective, the therapeutic interventions that improve the autonomic imbalance toward a more salubrious profile may serve to prevent or at least minimize the risk for cardiovascular diseases and death [79]. Given the intrinsic connection between the brain and the heart, a recent review pointed to cardiovascular and ANS activity, confirming a potential pathogenic brain–heart pathway to cardiovascular diseases [80].

HRV can be quantified by various methods: The HRV linear analysis was recently recommended for clinical practice and psychophysiological research [81]. Specifically, the linear (spectral) HRV short-term analysis allows for the faster isolation of the high-frequency respiratory-linked influences on HRV (HF-HRV: 0.15–0.4 Hz). In other words, the HF-HRV reflects physiological rapid HR oscillations according to breathing (i.e., HR increases during inspiration, and HR decreases during expiration)—respiratory sinus arrhythmia (RSA). Importantly, RSA is mediated mainly by the cardiac vagal nerve traffic originating in the nucleus ambiguous and therefore provides a non-invasive biomarker of cardiac vagal regulation indexed by the HF-HRV [82,83].

### 4.2. Blood Pressure Variability—An Index of Sympathetic Vascular Regulation in Anorexia Nervosa

While resting HR is predominantly under vagally-mediated cardiac control, the vascular tone is sympathetically regulated [84]. Mean arterial blood pressure (BP) as a critical hemodynamic factor depends on two hemodynamic parameters: cardiac output and total peripheral resistance; both parameters are under regulation and are mediated by the baroreflex mechanism and ANS. The deficiency of proper regulatory mechanisms included in the BP modulation can have important pathophysiological consequences, e.g., low BP results in inadequate blood flow to organs, resulting in syncope or shock, and high BP is associated with increased oxygen demand by the heart, ventricular remodelling, or vascular injury [84].

Blood pressure variability (BPV) is characterized by marked spontaneous oscillations over short-term or long-term time periods depending on the interplay of different cardiovascular control systems, such as the baroreceptor reflex, the vascular myogenic response, as well as changes in behavioural and emotional mechanisms [85]. The ability to monitor short-term instantaneous BP changes in time is based on the non-invasive “volume-clamp” method by Peňáz in the early 1970s [86,87]. Consequently, through the spectral analysis of the BP biosignal, information can be obtained about dominant sympathetically-mediated Mayer waves at a low-frequency band (LF-BPV: 0.04–0.15 Hz). Two basic mechanisms are responsible for these oscillations: central and baroreflex. While the autonomic oscillators within the central nervous system generate periodic fluctuations in autonomic nerve activity that are translated into corresponding oscillations in BP, influencing vascular sympathetic activity [88,89], the arterial baroreceptor reflex exhibits a resonance at the frequency of spontaneously occurring Mayer waves [88,89,90,91]. Thus, the index LF-BPV (especially systolic BP) is considered as a biomarker of sympathetic vascular regulation.

The arterial baroreflex plays a crucial role in short-term arterial BP control, haemodynamic stability and cardioprotection. The baroreflex evokes reciprocal responses of the ANS: When afferent baroreflex nerve traffic intensifies (this happens when BP increases), the efferent sympathetic traffic decreases while the efferent parasympathetic traffic increases, the inverse response occurs when BP decreases [84,92,93,94].

The arterial baroreflex is usually quantified by baroreflex sensitivity, which is defined as the change in the interbeat interval in milliseconds per unit change in BP (in mmHg). In this aspect, the baroreflex efferents to the sinoatrial node translate BP variability into HRV [84,92]. Furthermore, altered baroreflex sensitivity (BRS) contributes to the reciprocal reduction of parasympathetic activity and an increase of sympathetic activity—this shift in sympathovagal balance is associated with development and progression of cardiovascular diseases [95]. Thus, baroreflex sensitivity assessment is considered as a potential biomarker of proper cardiovascular reflex functioning, mediated by autonomic neural control between both parameters: HR and BP.

It is important to note that “cardiac baroreflex” and “sympathetic baroreflex” are different. Cardiac baroreflex is determined by assessment of the relationship of the RR intervals (i.e., HR) to a given change in arterial BP. The sympathetic baroreflex represents the relationship between diastolic arterial BP and vasoconstrictor sympathetic nerve activity (it is determined by muscle sympathetic activity) [84].

With respect to anorexia nervosa, several studies revealed insufficient sympathetic cardiovascular control [96,97]. Specifically, decreased mean BP (hypotension) is a typical finding for AN (e.g., review by Sachs et al. [68]). In this perspective, both sinus bradycardia and hypotension can have consequences in the maladaptive response to physiological load (e.g., orthostasis—posture change from lying to standing, in which haemodynamic adaptation is mediated through the baroreflex) resulting in cardiovascular adverse outcomes, such as a syncope. For example, the dramatic cardiovascular changes in AN have similar features to postural orthostatic tachycardia syndrome [68]. This assumption is confirmed by the studies revealing sympathetic hypofunction in both the resting and the standing positions in patients suffering from AN based on BPV analysis [96,97].

Additionally, a recent study found increased BRS associated with higher HRV, indicating an enhanced parasympathetic reflex of HR control in AN. This study referred to the BRS and the HRV as two major negative prognostic indicators of anorexia-associated arrhythmic death [98]. Notably, bradycardia—lower heart rate as a result of higher parasympathetic regulatory influences on the sinoatrial node in the heart—itself causes QT prolongation and increases the risk for the development of early after depolarizations, which are the causal event leading to torsades de pointes ventricular tachycardia and sudden death [99,100]. In this aspect, bradycardia associated with the presence of QT prolongation may result in increased risk of sudden cardiovascular death in anorexia nervosa [101,102]. Thus, we suggest that non-invasive biomarkers used for assessment of complex cardiovascular reflex control functioning could represent a highly sensitive methodological approach for early diagnosis and prevention of cardiovascular morbidity in AN.

Importantly, arterial properties evaluated using the carotid intima-media thickness, parameters of arterial stiffness, and endothelial function are interrelated with the autonomic regulation, particularly with sympathetic vascular control [103,104,105,106,107]. In this aspect, the studies on early atherosclerotic damage in patients with AN presented the evidence of endothelial dysfunction, selective peripheral vasoconstriction, and accelerated aortic arteriosclerosis [68,108,109,110]. Therefore, simultaneous assessment of these indices with the non-invasive autonomic biomarkers such as LF-BPV could offer clinically significant information about the cardiovascular risk associated with the effects of ANS dysregulation. 

Table 1 summarizes the latest studies regarding neuroendocrine, immune, and ANS dysregulation in anorexia nervosa.

## 5. Clinical Application

It is unknown whether the anorexia-linked cardiovascular dysregulation is reversible by specific treatment based on weight gain. Lachish et al. [123], based on the HRV changes in anorectic patients after short- and long-term weight gain, conclude that abnormal cardiac vagal control was presented not only in malnourished patients, but also persisted following short-term and long-term weight restoration. In contrast, a recent study referred to a shift toward parasympathetic dominance indicated by HRV parameters in children with AN that is improved after re-feeding, i.e., cardiovagal regulation indexed by HRV decreased. This effect could be explained by an increase of the intrinsic pacemaker rate as well as central–peripheral autonomic rapid response to the increased caloric intake during re-feeding [69]. Notably, re-feeding syndrome, which occurs during the first days of re-feeding, can be associated with a critically increased risk of acute, life-threatening cardiac complications [5].

It is interesting to note that restrained eating (i.e., intentional restriction of food intake to prevent weight gain or to promote weight loss) was associated with low cardiac vagal control [124]. As noted in a recent review [125], the cardiovagal regulation is positively related to interoceptive sensitivity [126], thus, the diet-related reductions in vagal modulation may diminish the ability to detect interoceptive signals such as information about the current homeostatic state [127,128], contributing to mental illnesses [129]. Thus, HRV may serve as a useful biomarker for identifying potentially beneficial or detrimental aspects of diet [125]. This issue is particularly important in prevention of the potential development of eating disorders including AN and related health complications as a result of life-style modifications (dietary regimen, excessive physical activity) during adolescence.

**Figure 2:**
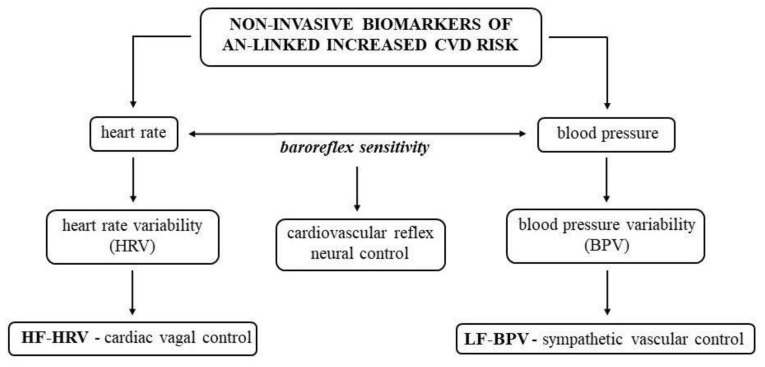
Potential non-invasive biomarkers for detection of the increased cardiovascular diseases (CVD) risk in anorexia nervosa (AN). HF-HRV, high frequency band of heart rate variability; LF-BPV, low frequency band of blood pressure variability.

Potential non-invasive biomarkers for detection of an AN-linked increase in risk of CVD are summarized in Figure 2.

## 6. Conclusions

Anorexia nervosa is associated with an altered neuroendocrine and inflammatory profile. Although most of these disturbances are reversible with appropriate treatment, several persistent neuroendocrine–immune alterations may contribute to the development and progression of AN and to increased AN-linked cardiovascular risk. Moreover, the complex assessment of cardiovascular neural control using non-invasive biomarkers could represent a promising tool for early diagnosis and personalized therapy in AN. Simultaneous evaluation of parameters of autonomic control could bring novel clinically important information about the mechanisms of increased risk of cardiovascular events in AN, especially in adolescence.

## Figures and Tables

**Table 1:** Recent studies of neuroendocrine, immune, and autonomic nervous system dysregulation in anorexia nervosa.

Recent Studies	Measured Parameters	Main Findings
Neuroendocrine dysregulation		
Het et al., 2020 [111]	Salivary cortisol and sAA were measured before, during, and after exposure to the Trier Social Stress Test pre- and post-treatment.	HPA hyporeactivity, blunted cortisol stress response associated with attenuated sAA levels at pre-treatment were found in ED patients compared to controls. After treatment, the blunted cortisol stress response persisted and sAA responses were normalized in ED patients.
Aulinas et al., 2020 [112]	Leptin, IGF-1, total T3, total T4, free T4 index, TSH, total T4/ total T3 ratio, and cortisol.	Serum leptin, IGF-1, total T3 levels, and total T3/total T4 ratio were significantly decreased in AN patients compared to that of controls.
Mancuso et al., 2020 [113]	Ghrelin, PYY, and BDNF levels were assessed before and after standardized breakfast.	Fasting ghrelin and PYY were higher and fasting BDNF was lower in AN patients compared to those of controls. After breakfast (over 120 min), ghrelin and PYY AUC were higher and BDNF AUC was lower in AN patients compared to those of controls.
Elegido et al., 2019 [114]	Leptin, soluble leptin receptor, adiponectin, and cortisol.	Leptin level was decreased, soluble leptin receptor, cortisol, and adiponectin levels were increased in AN patients compared to those of controls.
Paslakis et al., 2019 [115]	Ghrelin, leptin, cholecystokinin, PYY, adiponectin, and visfatin.	Leptin was significantly decreased and adiponectin significantly increased in AN patients compared to those of controls.
Podfigurna et al., 2018 [116]	Kisspeptin, FSH, LH, oestradiol, prolactin, testosterone.	Serum LH and oestradiol concentrations in AN patients were significantly lower compared to those of the control group.
Brambilla et al., 2018 [117]	GH and IGF-1.	GH was significantly increased and IGF-1 decreased in AN patients compared to those of controls.
Immune dysregulation		
Roczniak et al., 2020 [118]	IL-15.	Serum level of IL-15 was significantly higher in AN patients compared to that of controls.
Caroleo et al., 2019 [119]	IL-1α, IL-1β, IL-2, IL-4, IL-6, IL-8, IL-10, IFN-γ, TNF-α, MCP-1, VEGF, and EGF.	IL-1α, IFNγ, and IL-10 were significantly increased and EGF significantly decreased in AN patients compared to those of controls.
Tanaka et al., 2019 [120]	IL-18.	IL-18 was significantly decreased in AN patients compared to that of controls.
Elegido et al., 2019 [114]	IL-1β, IL-2, IL-6, and TNF-α.	Serum TNF-α and IL-2 showed significantly lower and higher values, respectively, in AN patients compared to those of controls.
Dalton et al., 2018 [121]	BDNF, bFGF, CRP, Eotaxin, Eotaxin-3, sFlt-1, GM-CSF, ICAM-1, IFNγ, IL-1α, IL-1β, IL-2, IL-4, IL-5, IL-6, IL-7, IL-8, IL-10, IL-12/IL-23p40, IL-12p70, IL-13, IL-15, IL-16, IL-17A, IP-10, MCP-1, MCP-4, MIP-1α, MIP-1β, PlGF, SAA, TARC, TYK2, TNF-α, TNF-β, VCAM-1, VEGF-A, VEGF-C, and VEGF-D.	IL-6, IL-15, and VCAM-1 concentrations were significantly elevated and concentrations of BDNF, TNF-β, and VEGF-A were significantly lower in AN patients compared to those of controls.
Autonomic nervous system dysregulation		
Het et al., 2020 [111]	HR and HF-HRV were measured before, during, and after exposure to the Trier Social Stress Test at pre- and post-treatment.	ED patients showed significantly lower HR and higher HF-HRV before treatment compared to those of controls. These changes were reversible after treatment.
Tonhajzerova et al., 2020 [110]	HRV and BPV.	LF-BPV was significantly lower in AN adolescents compared to that of controls, indicating insufficient sympathetic cardiovascular control in anorexia nervosa already at adolescent age.
Billeci et al., 2019 [122]	HR and HRV indices were measured at baseline, during light physical exercise, and during recovery.	HR, LF-HRV, and the LF/HF ratio were significantly lower, while SDNN, RMSSD, and HF-HRV were significantly higher in the AN group compared to those of controls at baseline. During light physical exercise, HR, LF-HRV, and the LF/HR ratio significantly increased followed by significant decrease at recovery among the AN group. The opposite trend was found for LF-HRV and HF-HRV associated with no change in the LF/HF ratio in controls. The AN group showed no significant changes in SDNN and RMSSD in contrast to those values in the control group (increased SDNN, RMSSD, during light physical activity followed by a decrease at recovery).

AN, anorexia nervosa; AUC, area under the curve; BDNF, brain-derived growth factor; bFGF, basic fibroblast growth factor; BPV, blood pressure variability; CRP, C-reactive protein; ED, eating disorders; EGF, epidermal growth factor; FSH, follicle-stimulating hormone; GH, growth hormone; GM-CSF, granulocyte-macrophage colony-stimulating factor; HF-HRV, high frequency band of heart rate variability; HPA, hypothalamic–pituitary–adrenal; HR, heart rate; HRV, heart rate variability; ICAM, intercellular adhesion molecule; IFN, interferon; IGF-1, insulin-like growth factor; IL, interleukin; IP, interferon-induced protein; LF-BPV, low frequency band of blood pressure variability; LF-HRV, low frequency band of heart rate variability; LH, luteinizing hormone; MCP, monocyte chemoattractant protein; MIP, macrophage inflammatory protein; PlGF, placental growth factor; PYY, peptide YY; RMSSD, root mean square of successive differences; sAA, salivary alpha-amylase; SAA, serum amyloid A; SDNN, standard deviation of the NN intervals; sFlt-1, fms-like tyrosine kinase-1; T3, triiodothyronine; T4, thyroxine; TARC, thymus and activation-regulated chemokine; TNF, tumor necrosis factor; TSH, thyroid-stimulating hormone; TYK2, tyrosine kinase-2; VCAM, vascular cell adhesion protein; VEGF, vascular endothelial growth factor.

## Abbreviations

AN	Anorexia nervosa
ANS	Autonomic nervous system
HPA	Hypothalamic–pituitary–adrenal
CRH	Corticotropin-releasing hormone
ACTH	Adrenocorticotropic hormone
HPG	Hypothalamic–pituitary–gonadal
FHA	Functional hypothalamic amenorrhea
GnRH	Gonadotropin-releasing hormone
FSH	Follicle stimulating hormone
LH	Luteinizing hormone
CVD	Cardiovascular disease
HPT	Hypothalamic–pituitary–thyroid
T3	Triiodothyronine
T4	thyroxine
GH	Growth hormone
IGF-1	Insulin-like growth factor 1
PYY	Peptide YY
BMI	Body mass index
IFN	Interferon
IL	Interleukin
TNF	Tumour necrosis factor
NTS	Nucleus tractus solitarii
α7nAChRs	α7 subunits of containing acetylcholine receptors
HR	Heart rate
HRV	Heart rate variability
CAN	Central autonomic network
HF-HRV	High frequency band of heart rate variability
RSA	Respiratory sinus arrhythmia
BP	Blood pressure
BPV	Blood pressure variability
LF-BPV	Low frequency band of blood pressure variability
BRS	Baroreflex sensitivity
